# An artifıcial ıntelligence approach to automatic tooth detection and numbering in panoramic radiographs

**DOI:** 10.1186/s12880-021-00656-7

**Published:** 2021-08-13

**Authors:** Elif Bilgir, İbrahim Şevki Bayrakdar, Özer Çelik, Kaan Orhan, Fatma Akkoca, Hande Sağlam, Alper Odabaş, Ahmet Faruk Aslan, Cemre Ozcetin, Musa Kıllı, Ingrid Rozylo-Kalinowska

**Affiliations:** 1 Department of Oral and Maxillofacial Radiology, Faculty of Dentistry, Eskisehir Osmangazi University, Eskişehir, Turkey; 2 Department of Mathematics-Computer, Faculty of Science, Eskisehir Osmangazi University, Eskisehir, Turkey; 3 Department of Oral and Maxillofacial Radiology, Faculty of Dentistry, Ankara University, Ankara, Turkey; 4 Private Practice, Eskisehir, Turkey; 5 Faculty of Dentistry, Eskisehir Osmangazi University, Eskişehir, Turkey; 6 Department of Dental and Maxillofacial Radiodiagnostics, Medical University of Lublin, ul. Doktora Witolda Chodźki 6, 20-093 Lublin, Poland; 7 Center of Research and Application for Computer Aided Diagnosis and Treatment in Health, Eskisehir Osmangazi University, Eskisehir, Turkey

**Keywords:** Artificial intelligence, Deep learning, Tooth, Panoramic radiography

## Abstract

**Background:**

Panoramic radiography is an imaging method for displaying maxillary and mandibular teeth together with their supporting structures. Panoramic radiography is frequently used in dental imaging due to its relatively low radiation dose, short imaging time, and low burden to the patient. We verified the diagnostic performance of an artificial intelligence (AI) system based on a deep convolutional neural network method to detect and number teeth on panoramic radiographs.

**Methods:**

The data set included 2482 anonymized panoramic radiographs from adults from the archive of Eskisehir Osmangazi University, Faculty of Dentistry, Department of Oral and Maxillofacial Radiology. A Faster R-CNN Inception v2 model was used to develop an AI algorithm (CranioCatch, Eskisehir, Turkey) to automatically detect and number teeth on panoramic radiographs. Human observation and AI methods were compared on a test data set consisting of 249 panoramic radiographs. True positive, false positive, and false negative rates were calculated for each quadrant of the jaws. The sensitivity, precision, and F-measure values were estimated using a confusion matrix.

**Results:**

The total numbers of true positive, false positive, and false negative results were 6940, 250, and 320 for all quadrants, respectively. Consequently, the estimated sensitivity, precision, and F-measure were 0.9559, 0.9652, and 0.9606, respectively.

**Conclusions:**

The deep convolutional neural network system was successful in detecting and numbering teeth. Clinicians can use AI systems to detect and number teeth on panoramic radiographs, which may eventually replace evaluation by human observers and support decision making.

## Background

Panoramic radiography is a method for producing a single tomographic image of the facial structures that includes both the maxillary and mandibular dental teeth and their supporting structures. It has a number of advantages, including a relatively low radiation dose, short imaging time, and low burden to the patient. Panoramic radiography is widely used in clinical settings to solve diagnostic problems in dentistry because it shows both jaws in a single image and allows evaluation of all teeth. The automated diagnosis of panoramic radiographs could help clinicians in daily practice [1, 2].

Artificial intelligence (AI) is defined as the ability of a machine to mimic intelligent behavior to perform complex tasks, such as decision making, recognition of words and objects, as well as problem solving. Deep learning systems are among the most promising systems in AI. The deep learning approach has been elaborated to improve the performance of traditional artificial neural networks (ANNs) using complex architectures. Deep learning methods are characterized by multiple levels of representation, and the raw data are processed to yield classification or performance of detection tasks [3–5]. In deep learning, multiple layers of algorithms are classified into conjunct and important hierarchies to provide meaningful data. These layers collect input data and provide output that undergoes gradual changes as the AI system learns new features based on the supplied data. ANNs are composed of thousands to millions of connected nodes or units. Links between the nodes or units are activated, and the activation spreads from one unit to another, with each link activation weighted with a numerical value that determines the strength of the link [3]. ANNs should be trained using educational data sets in which the initial image data sets should be manually tagged by the algorithm to suit the ground truth [3, 6, 7].

Convolutional neural networks (CNNs) have been applied in computerized vision applications within ANNs. In this deep ANN class, convolution operations are used to obtain feature density maps. In such maps, the density of each pixel/voxel is the sum of the pixels/voxels in the original image and the sum of pixels/voxels with convolution matrices also bearing the name of the nucleus. Specific tasks, such as sharpening, blurring, or edge detection, are performed by various cores. CNNs are inspired by nature and mimic the behavior of the complex structures of the brain cortex, in which small regions of the visual field are analyzed by cells sensitive to this area. The deep CNN architecture allows complex features to be decoded from simple features based on raw image data, thus achieving redundancy in identification of specific features [8–12].

AI became a cornerstone of radiology with the introduction of a digital picture archiving and communication system that provided large amounts of imaging data, offering great potential for AI training [8, 13, 14]. However, the use of AI, especially CNNs, in dentistry has been limited. Panoramic radiography is a baseline imaging modality and an essential tool for diagnosis, treatment planning, and follow-up in dentistry. Therefore, in this study, it was considered worthwhile to train a deep CNN for detection and numbering of teeth and to compare the performance of the CNN with that of expert human observers.

## Methods

### Patient selection

This retrospective observational study evaluated a radiographic data set that included 2482 anonymized panoramic radiographs from adults obtained between January 2018 and January 2020 from the archive of Eskisehir Osmangazi University, Faculty of Dentistry, Department of Oral and Maxillofacial Radiology. Panoramic radiographs with artifacts related to metal superposition, position errors, movement, etc., were removed from the data set. Panoramic radiographs showing developmental anomalies, crowded teeth, malocclusion, rotated teeth, anterior teeth with inclination, retained deciduous and supernumerary teeth, and transposition between the upper left canine and lateral incisor were excluded from the study. Panoramic radiographs showing teeth with dental caries, restorative fillings, crowns and bridges, implants, etc., were included in the study. The non-interventional Clinical Research Ethical Committee of Eskisehir Osmangazi University approved the study protocol (decision date and approval number: 06.08.2019/14). The study was conducted in accordance with the principles of the Declaration of Helsinki.

### Radiographic data set

All panoramic radiographs were obtained using the Planmeca Promax 2D (Planmeca, Helsinki, Finland) panoramic dental imaging unit with the following parameters: 68 kVp, 16 mA, 13 s.

### Image evaluation

Two oral and maxillofacial radiologists (E.B. and I.S.B.) with 10 years of experience and an oral and maxillofacial radiologist (F.A.K.) with 3 years of experience provided ground truth annotations for all images using Colabeler (MacGenius, Blaze Software, CA, USA). Annotations were collected by asking the experts to draw bounding boxes around all teeth and at the same time to provide a class label for each box with the tooth number according to the FDI tooth numbering system.

### Deep convolutional neural network

An arbitrary sequence was generated using the open-source Python programming language (Python 3.6.1, Python Software Foundation, Wilmington, DE, USA; retrieved August 01, 2019 from https://www.python.org). Inception v2 Faster R-CNN (region-based CNN) network implemented with TensorFlow library was used to create a model for tooth detection and numbering. This method consists of 22 deep layers, which can obtain different scale features by applying various sizes of convolutional filters within the same layer. It includes an auxiliary classifier, totally interconnected layers, and a total of nine start modules, including softmax functions [15].

### Model pipeline

**Fig. 1:**
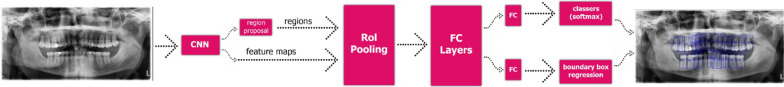
System architecture and tooth detection and numbering pipeline

In this study, we developed an AI algorithm (CranioCatch, Eskisehir, Turkey) to automatically detect and number teeth using deep learning techniques, including Faster R-CNN Inception v2 models, on panoramic radiographs. Using the Inception v2 architecture as transfer learning, the transfer values in the cache were first saved, and then a fully connected layer and softmax classifier was used to build the final model layers (Fig. 1). Training was performed using 7000 steps on a PC with 16 GB RAM and the NVIDIA GeForce GTX 1050 graphics card. The training and validation data sets were used to predict and generate optimal CNN algorithm weight factors.

Before training, each radiograph was resized from the original dimensions of 2943 × 1435 pixels to 1024 × 512 pixels. The training data set in which 32 different teeth were labeled at the same time consisted of 1984 images. Among the teeth on the 1984 panoramic radiographs in the training group, 56,251 teeth were labeled. The classes were as follows: 11-12-13-14-15-16-17-18-21-22-23-24-25-26-27-28-31-32-33-34-35-36-37-38-41-42-43-44-45-46-47-48. To compensate for the tooth numbers that could not be found, the order of the teeth was made using the single tooth detection model. The gap between the right and left teeth was checked using a single tooth detection model for missing teeth, and the gap was filled.

### Training phase

The images were divided into training (80%), validation (10%), and test (10%) groups. For each quadrant (regions 1, 2, 3, and 4), 1984, 249, and 249 images were randomly distributed into the training, test, and validation groups, respectively.

**Fig. 2:**
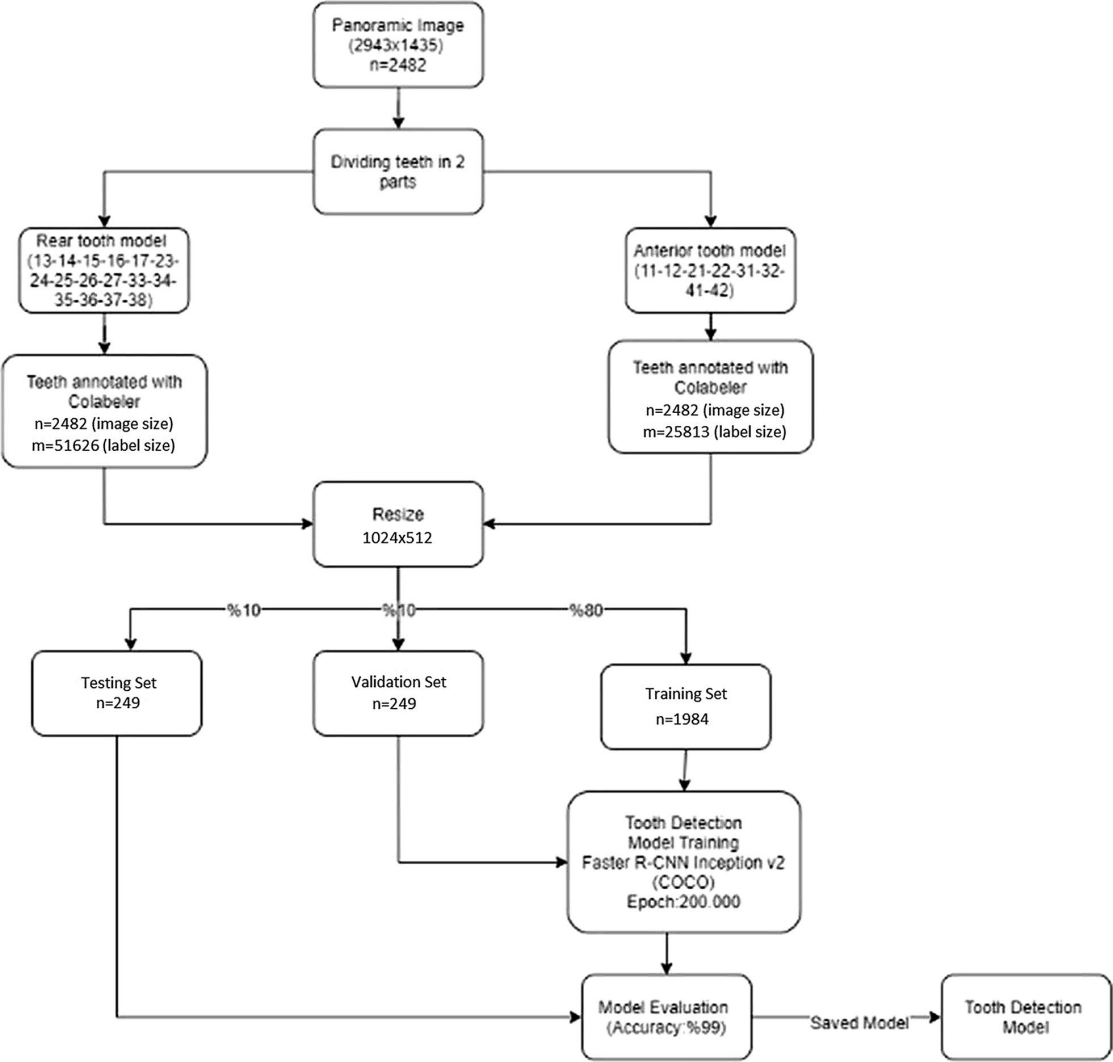
The diagram of Dental Object Detection Model (CranioCatch, Eskisehir-Turkey)

**Fig. 3:**
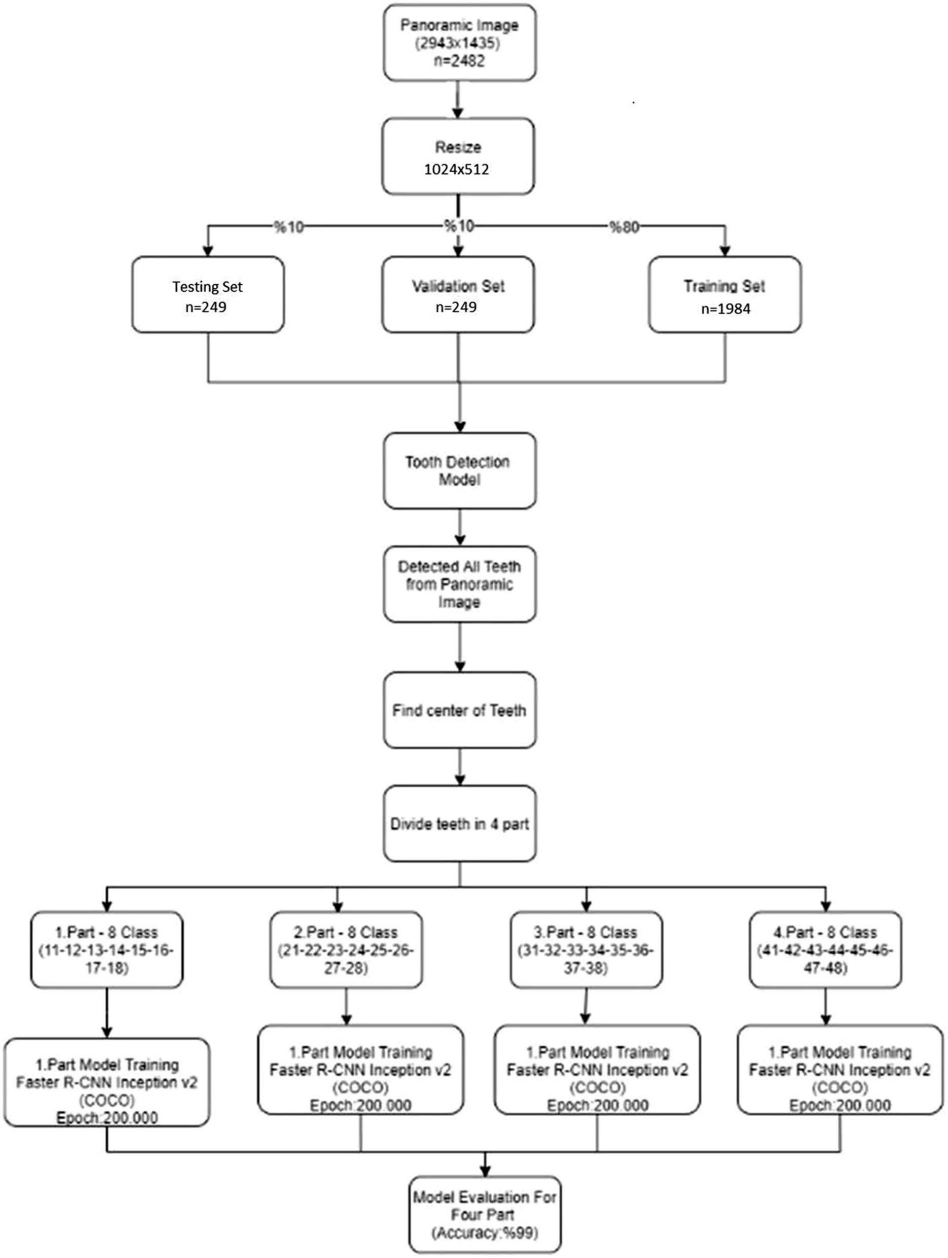
The diagram of Different Models (CranioCatch, Eskisehir-Turkey)

**Fig. 4:**
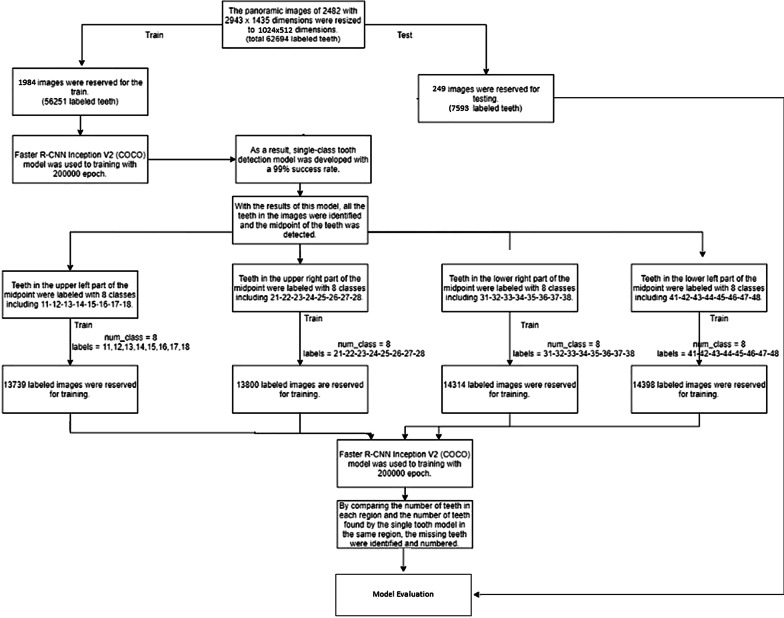
The diagram of AI model (CranioCatch, Eskisehir-Turkey) developing stages

The CranioCatch approach for detecting teeth is based on a deep CNN using 200,000 epochs trained with faster R-CNN inception v2 with a learning rate of 0.0002. At this point, the exact type of tooth must be specified using a separate deep CNN. Once trained, the model is utilized to identify the presence of teeth in the following manner: dental object detection model; different models (100%), 11-12-21-22-31-32-41-42 numbered teeth were detected using the anterior tooth model, and the remaining numbered teeth were detected using the rear tooth model; jaw midpoint detection; upper/lower jaw classification model (100%); training in four models (Figs. 2, 3, 4).

### Statistical analysis

The confusion matrix, a useful table that summarizes the predicted and actual situations, was used as a metric to calculate the success of the model. The following procedures and metrics were used to assess the success of the AI model:

Initially, true positive (TP), false positive (FP), and false negative (FN) rates were calculated.

**TP**: the outcome in which the model correctly predicts the positive class (teeth correctly detected and numbered on panoramic radiographs).

**FP**: the outcome in which the model incorrectly predicts the positive class (teeth correctly detected but incorrectly numbered on panoramic radiographs).

**FN:** the outcome in which the model incorrectly predicts the negative class (teeth incorrectly detected and numbered on panoramic radiographs).

The following metrics were then calculated using the TP, FP, and FN values:

- **Sensitivity (Recall):** TP/(TP + FN)
- **Precision:** TP/(TP + FP)
- **F1 Score:** 2TP/(2TP + FP + FN)
- **False Discovery Rate = **FP/(FP + TP)
- **False Negative Rate = **FN/(FN + TP)

## Results

**Fig. 5:**
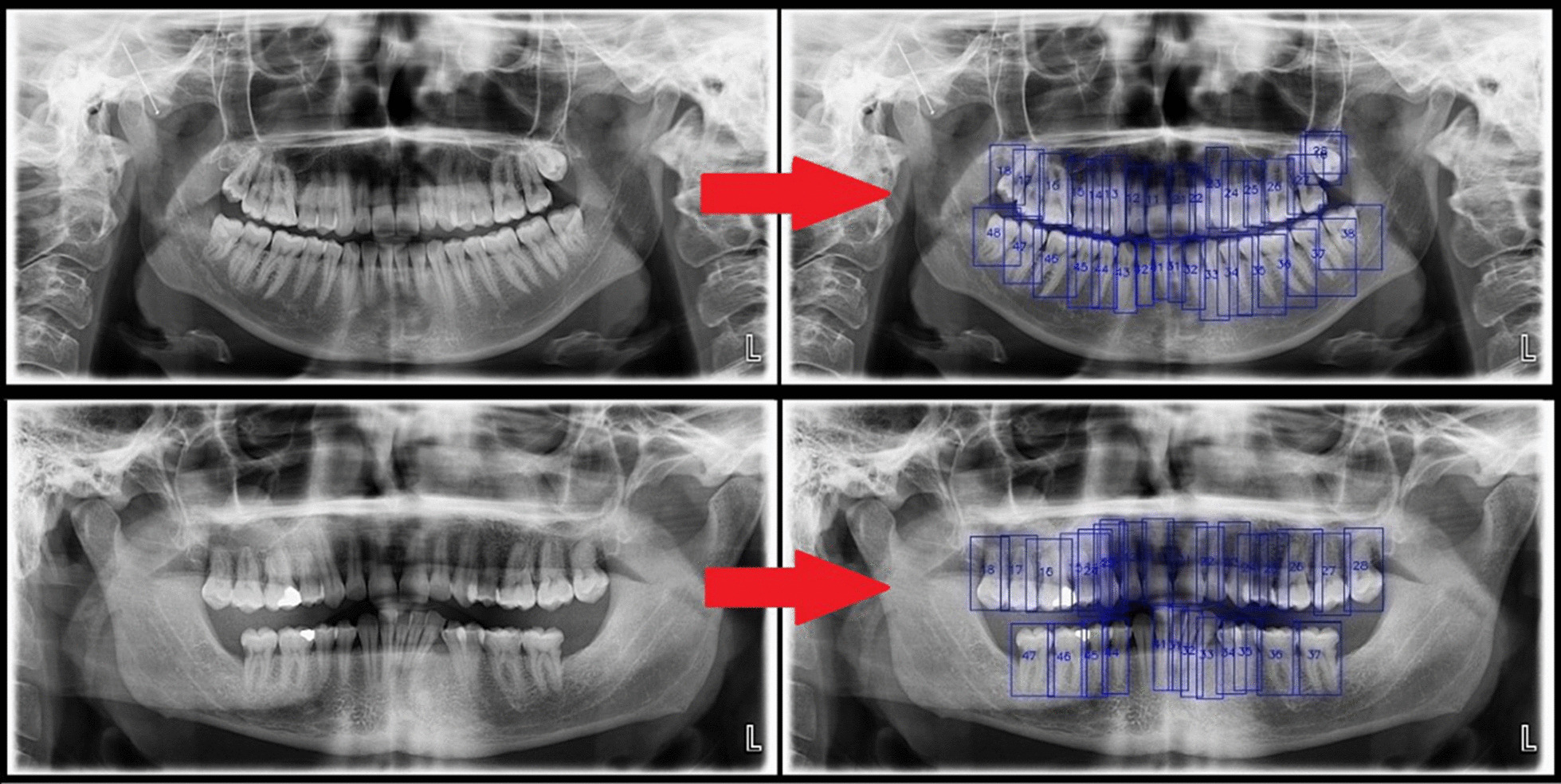
Detecting and numbering the teeth with the deep convolutional neural network system in panoramic radiographs

The deep CNN system was successful in detecting and numbering the teeth (Fig. 5). The numbers of TP, FP, and FN results were 1388, 50, and 64 in all quadrants. The sensitivity and precision rates were promising for detecting and numbering the teeth. Consequently, the estimated sensitivity, precision, false discovery rate, false negative rate, and F-measure were 0.9559, 0.9652, 0.0348, 0.0441, and 0.9606, respectively (Tables 1, 2).

**Table 1:** The number of teeth correctly and incorrectly detected and numbered by the AI model in terms of the region

Quadrant	Region-1	Region-2	Region-3	Region-4	Total
True positives (TP)	1710	1610	1800	1820	6940
False positives (FP)	85	60	70	35	250
False negatives (FN)	95	160	20	45	320

**Table 2:** The value of AI model estimation performance measure using confusion matrix

Measure	Value	Derivations
Sensitivity (Recall)	0.9559	TPR = TP/(TP + FN)
Precision	0.9652	PPV = TP/(TP + FP)
False Discovery Rate	0.0348	FDR = FP/(FP + TP)
False Negative Rate	0.0441	FNR = FN/(FN + TP)
F1 Score	0.9606	F1 = 2TP/(2TP + FP + FN)

## Discussion

Radiology has undergone two major digital revolutions with the introduction of advanced imaging methods and the development of workstations and archiving systems. These developments have been followed by the use of AI especially in radiographic analysis [8, 13]. The main advantage of machine learning, as radiologists are trained to evaluate medical images repeatedly, is that the dedicated AI model can develop and learn with experience due to increased training based on large and new image data sets. Using AI diagnostic models, radiologists hope not only to read and report many medical images but also to improve work efficiency and obtain more precise results in the accurate diagnosis of various diseases [8, 9].

The application of AI in dentistry is a relatively new development. Deep learning and deep CNNs have been applied to the detection of objects, periapical lesions, caries, and fracture determination [11, 12, 16–21]. There have also been studies of the application of AI to cone-beam CT, panoramic, bitewing, and periapical radiographs for charting purposes [9–11, 22–24]. Miki et al. [23] applied an automated method for classifying teeth in cone-beam CT images based on a deep CNN. They reported an accuracy of dental charting by deep CNN of 91.0% and concluded that AI could be an efficient tool for automated dental charting and in forensic dentistry [23]. Zhang et al. [25] described the use of a label tree with a cascade network for effective recognition of teeth. They applied the deep learning technique not only to tooth detection but also classification of dental periapical radiographs. The performance of the technique was good even with limited training data, with a precision of 95.8% and recall of 96.1% [25]. Lin et al. [22] developed a dental classification and numbering system that could be applied to intraoral bitewing radiographs for segmentation, classification, and numbering of teeth. The bitewing radiographs were processed using homomorphic filtering, homogeneity-based contrast stretching, and adaptive morphological transformation to improve the contrast and evenness of illumination, and they reported a numbering accuracy of 95.7% [22]. Chen et al. [26] presented a deep learning approach for automated detection and numbering of teeth on dental periapical films. They reported that both precision and recall exceeded 90%, and the mean value of the IOU [intersection over union] between detected boxes and ground truth also reached 91%. In addition, the results of the machine learning system were similar to those obtained by junior dentists [26]. Tuzoff et al. [24] focused on tooth detection and numbering on panoramic radiographs using a trained CNN-based deep learning model to provide automated dental charting according to the FDI two-digit notation. They aimed to achieve numbering of maxillary and mandibular teeth in a single image and used the state-of-the-art Faster R-CNN model based on the VGG-16 convolutional architecture. Their results were promising and showed that AI deep learning algorithms have the potential for practical application in clinical dentistry [24]. VGG-16 consists of 16 layers with two convolution layers: a pooling layer and a fully connected layer. The aim of the VGG network is to develop a much deeper architecture with much smaller filters. Inception V2 used in this study consists of 22 deep layers with convolutional filters of various sizes in the same layer. VGG-16 and Inception V2 achieved top-1 classification accuracy on Image-Net of 71% and 73.9%, respectively [27].

Consistent with the literature, the precision and sensitivity obtained in our research were high. The relatively low values obtained by studies performed on periapical and bitewing radiographs can be explained by the fact that the system first determines whether the graph belongs to the upper or lower jaw, whereas CNN-based deep learning is performed in fewer steps on panoramic radiographs. Although the present algorithm based on the Faster R-CNN showed promising results, this study had some limitations. Further studies are needed to determine the estimation success of each type of tooth (incisors, canines, premolars, molars) using larger data sets, as well as to examine additional types of CNN architectures and libraries. An improved method may be developed with better tooth detection and numbering results. Future studies should also investigate the possible advantages of using an AI on radiographic images with lower radiation doses.

## Conclusion

In the present study, a deep CNN was proposed for tooth detection and numbering. The precision and sensitivity of the model were high and showed that AI is useful for tooth identification and numbering. This AI system can be used to support clinicians in detecting and numbering teeth on panoramic radiographs and may eventually replace evaluation by human observers and improve performance in the future.

## Abbreviations

AI: Artificial intelligence
ANNs: Artificial neural networks
CNN: Convolutional Neural Network
CT: Computed tomography
FN: False negative
FP: False positive
HOG: Histogram of oriented gradients
PACS: Picture archiving and communication system
SIFT: Scale-invariant feature transform
TP: True positive

## Declarations

### Availability of data and materials

The datasets used and/or analyzed during the current study are available from the corresponding author on reasonable request.
